# The Breast Milk Immunoglobulinome

**DOI:** 10.3390/nu13061810

**Published:** 2021-05-26

**Authors:** Karla Rio-Aige, Ignasi Azagra-Boronat, Margarida Castell, Marta Selma-Royo, María Carmen Collado, María J. Rodríguez-Lagunas, Francisco J. Pérez-Cano

**Affiliations:** 1 Physiology Section, Department of Biochemistry and Physiology, Faculty of Pharmacy and Food Science, University of Barcelona (UB), 08028 Barcelona, Spain; rioaigekarla@ub.edu (K.R.-A.); ignasiazagra@ub.edu (I.A.-B.); margaridacastell@ub.edu (M.C.); mjrodriguez@ub.edu (M.J.R.-L.); 2 Nutrition and Food Safety Research Institute (INSA-UB), 08921 Santa Coloma de Gramenet, Spain; 3 Institute of Agrochemistry and Food Technology-National Research Council (IATA-CSIC), 46890 Paterna, Valencia, Spain; mselma@iata.csic.es (M.S.-R.); mcolam@iata.csic.es (M.C.C.)

**Keywords:** immunoglobulin, IgA, breast milk, immunoglobulinome

## Abstract

Breast milk components contribute to the infant’s immune development and protection, and among other immune factors, immunoglobulins (Igs) are the most studied. The presence of IgA in milk has been known for a long time; however, less information is available about the presence of other Igs such as IgM, IgG, and their subtypes (IgG1, IgG2, IgG3, and IgG4) or even IgE or IgD. The total Ig concentration and profile will change during the course of lactation; however, there is a great variability among studies due to several variables that limit establishing a clear pattern. In this context, the aim of this review was firstly to shed light on the Ig concentration in breast milk based on scientific evidence and secondly to study the main factors contributing to such variability. A search strategy provided only 75 studies with the prespecified eligibility criteria. The concentrations and proportions found have been established based on the intrinsic factors of the study—such as the sampling time and quantification technique—as well as participant-dependent factors, such as lifestyle and environment. All these factors contribute to the variability of the immunoglobulinome described in the literature and should be carefully addressed for further well-designed studies and data interpretation.

## 1. Introduction

### 1.1. Breast Milk: A Source of Immunomodulatory Components

Breast milk is the biological fluid produced by the mother’s breasts of mammalians in order to nourish infants and also to confer on them protection from disease until their own immune system matures [1]. Accordingly, the World Health Organization (WHO) recommends exclusive breastfeeding for the first 6 months of life, followed by continued breastfeeding with adequate complementary foods for up to 2 years or beyond, as mutually desired by mother and infant [2]. Breast milk has been tailored during human evolution to meet the demands of the infant. Its composition varies within feeds, during the day, and between mothers [3]. Interindividual variability has been attributed to genetic variation, maternal adiposity, and nutrition, among other factors [4,5,6]. The composition of human milk is dynamic and changes throughout lactation. The first form of milk produced by the mammary glands during the first 2–4 days after delivery is colostrum, which is produced in low volumes (300–400 mL/day) and has higher levels of protein and lower levels of carbohydrates and fat content than mature breast milk. Moreover, colostrum is richer in immunological components, such as immunoglobulins (Igs), lactoferrin, leucocytes, and oligosaccharides, suggesting that its primary functions are immunological rather than nutritional [3,7]. From days 4–5 after delivery, colostrum changes to transition milk, which is characterized by a higher yield (500–800 mL/day) and by lower protein and Ig content, accompanied by an increase in lactose, fat, and water-soluble vitamins to meet the growth demands. Finally, mature milk remains relatively similar in composition 6 weeks after delivery [3,8]. While 87% of breast milk is water, the remaining 13% is nutritional components and bioactive compounds that have beneficial non-nutritional functions [9]. These latter compounds include a wide range of antimicrobial factors, microorganisms, cytokines, hormones, growth modulators, and digestive enzymes, among others, although the Igs are of special relevance for the baby’s immune protection and development [10]. 

### 1.2. The Mammary Gland as a Source of Protective Immunoglobulins for the Newborn

**Figure 1:**
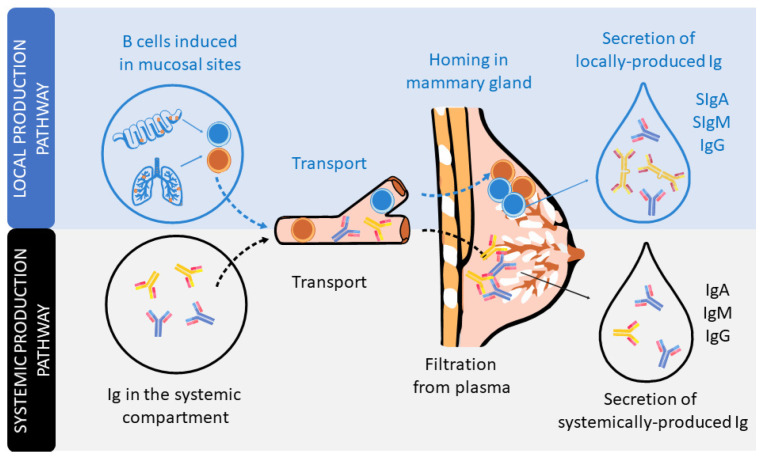
The secretion of Igs in human milk. Schematic figure of the local production pathway of Igs (involving the B cell homing to the mammary gland and participation of the secretory component) and the systemic production pathway (involving the monomeric Igs plasma filtration from plasma).

In humans and non-human primates, the transplacental transfer of immunoglobulins (Igs) from the mother to the fetus provides passive immunization to the offspring before delivery. However, it is after delivery when, in many animals, such as rodents or pigs, the Igs present in colostrum, the first breast milk produced, can be absorbed in the small intestine towards the systemic circulation. However, this phenomenon, very well described in pigs and rodents, is rather limited in humans, in which absorption of trace amounts of Ig can be negligible [11,12,13]. From this perspective, the existence of Igs in human breast milk has long been known [14]. The type, structure, and concentration of these Igs differ from those found in plasma [15,16]. Indeed, the Ig composition of breast milk arises from Igs produced locally in the mammary gland or transferred from the plasma (Figure 1). 

The dominant Ig in human milk is a special form of IgA, secretory IgA (SIgA), which is common to all mucosal secretions. This particular structure has multiple features and functions that make it optimal for mucosal defense, such as being able to neutralize pathogens before they come into contact with epithelial cells, being highly resistant and stable, and also preventing excessive inflammation or damage to the tissues [17,18,19]. The production of SIgA is induced by pathogens or commensal microorganisms found in mucosal sites after triggering T-helper (Th) and natural killer (NK)-T cells (T-dependent) or innate cells, such as lymphoid cells (ILCs) or plasmacytoid dendritic cells (pDCs) (T-independent). In particular, the switch from IgM+ B lymphocytes to IgA+ B lymphocytes is mainly driven by the transforming growth factor (TGF)-β and cytokines produced by Th2 cells, including interleukin (IL-4), IL-5, IL-6, IL-10, and IL-21 [11,18,20]. It is important to highlight that during the pregnancy period, in order to provide a maternal immune tolerance environment, the ratio of pro- and anti-inflammatory cytokines, related to Th1 and Th2 cells, respectively, is polarized towards a Th2 response. Moreover, this dominance of the Th2 response persists for some months in the neonate, during the lactation period, before reaching the Th1/Th2 equilibrium [21,22,23].

IgA-producing cells in the mammary gland arise from mucosal tissues mainly found in both the gut and airways (Figure 1). The migration of B cells takes place due to their expression of the chemokine receptor (CCR)-10, which binds to the chemokine ligand (CCL)-28 expressed in the mammary gland [24]. IgA is produced in dimers, containing a polypeptide called the J chain, which is excreted by secretory mammary cells. This transport is mediated by the polymeric Ig receptor (pIgR), also termed the secretory component (SC). The pIgR is cleaved after transcytosis and partly remains in the IgA dimer, constituting SIgA antibodies in the breast milk [17]. In addition, breast milk also contains secretory IgM (SIgM), IgM, and IgG antibodies, but in smaller proportions. Like SIgA, there is selective homing to the mammary gland of plasma cells that produce IgM and IgG, which are subsequently transported into breast milk through pIgR. In addition, pIgR can also transport Igs found in serum [15,16].

With regard to functionality, it has been proven that IgA induces tolerance to microbial and food antigens in both mice and human neonates [25,26,27,28]. However, it has also been demonstrated that milk IgG immune complexes are necessary for tolerance induction in mice [29,30]. IgM and IgG—mainly IgG1 and IgG3 in humans [31]—activate the complement pathway for pathogen clearance and initiation of innate response [32]. Commensal-specific IgG and IgA from maternal milk are very important to dampen T-dependent immune responses against commensal microbiota in neonates [33]. With respect to IgG4, it is the least abundant subclass of IgG in human breast milk and serum [34]. However, it increases in allergen response [31] and has anti-inflammatory properties, since it inhibits immune precipitation and complement activation [35]. Therefore, IgG4 is part of the Th2 response [36,37]. IgG2 is well known for having an important role in the defense against bacterial capsular polysaccharide antigens [31] and for its low capacity to activate the complement system [31,36]. It is thought that IgG2 is involved in Th1 response (IgG1 + IgG2 + IgG3), but this is not yet firmly established [36,37]. Moreover, there are studies that report that IgG2, in addition to IgG4, has a low inflammatory potential at intestinal level [38]. IgE is also present in breast milk, but its functions in neonates are still controversial [39]. Furthermore, its levels in childhood seem to depend on maternal IgE concentration [39]. Moreover, allergen-specific IgE and IgG can be transmitted from maternal blood to human breast milk [40]. 

### 1.3. Do We Really Know the Immunoglobulin Concentration in Milk?

Many studies have described the Ig levels in breast milk, mainly IgA, but the other Igs have been less studied. The composition of Igs in milk has been addressed from different perspectives, such as population, including geography, genetics, and diet, as well as taking into account different gestational and delivery factors (antibiotics, gestational age, type of delivery), different collection time points (colostrum, transition, and mature milk), and the use of different techniques (ELISA, single radial immunodiffusion, radioimmunoassay, beads). In addition, some of these determinations were performed in a low number of samples. All these factors could explain why IgA concentration in a particular study can differ by up to 50 times from others [41,42,43,44,45]. Thus, comparison among studies is not an easy task. Although some literature exists, less information is available regarding the IgM, IgG, and IgG subclasses, and even less for IgE or IgD, again with the above limitations. Therefore, the aim of this review was firstly to shed light on the Ig concentration in breast milk and secondly to study the main factors influencing such variability.

## 2. Materials and Methods

**Figure 2:**
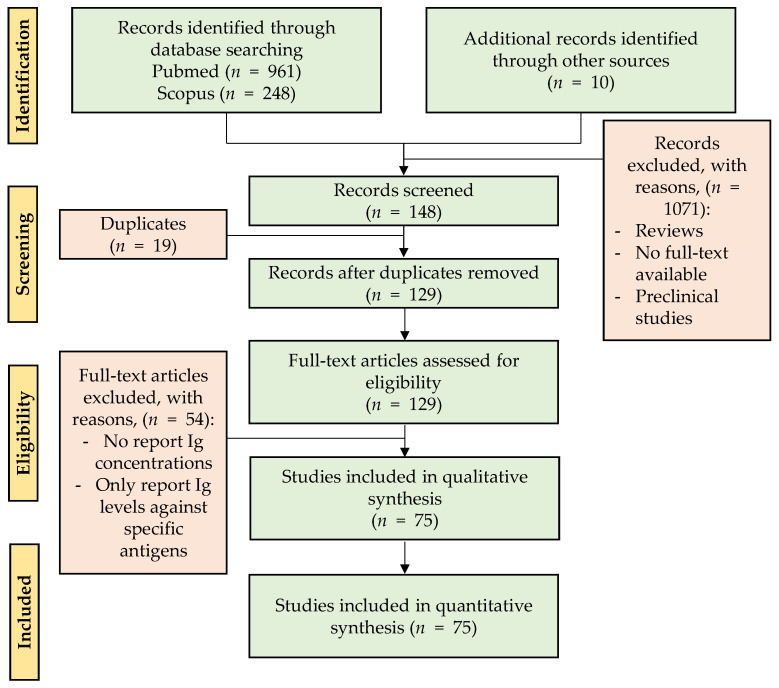
Diagram of the different phases of the search strategy. The flow figure shows the number of articles obtained after the identification and screening steps and those finally used for the review after the eligibility and final inclusion steps. Other sources correspond to the articles related to the topic found in the bibliography of the revised documents.

A total of 1209 articles were retrieved (up to October–November 2020) following the Preferred Reporting Items for Systematic Review and Meta-Analysis (PRISMA) in the PubMed and Scopus databases by using different groups of keywords in order to find searches related to a larger number of articles. The definitive keywords were “Immunoglobulins(Title/Abstract) AND (breast milk(Title/Abstract) OR human milk OR colostrum)(Title/Abstract)” in PubMed, and TITLE ((human AND milk OR colostrum) AND iga OR siga) OR TITLE ((human AND milk OR colostrum) AND igm OR sigm) OR TITLE ((human AND milk OR colostrum) AND igg) OR TITLE ((human AND milk OR colostrum) AND ige) OR TITLE ((human AND milk OR colostrum) AND igd) OR TITLE ((human AND milk OR colostrum) AND sc) OR TITLE ((human AND milk OR colostrum) AND immunoglobulins)“ in Scopus, without restriction by publication year. Abstracts or full texts were read and submitted to the established flow selection process shown in Figure 2.

Those articles from the SCOPUS and PubMed searches and from other additional sources (i.e., articles related to the topic found in the bibliography of the revised documents) that did not meet the inclusion criteria were excluded. In total, 1090 studies were excluded because they were reviews, duplicates, or preclinical studies or because the full text was not available on the Internet. Thus, review articles were filtered from the search and only used to support the findings, but not as a primary source. A subtotal of 129 records were included in the eligibility phase and, after reading the full texts, those that did not report Ig concentrations in measure of centrality and spread were excluded; thus, 75 studies were included in the qualitative synthesis. 

The information extracted from the studies was as follows: year of publication, population characteristics, sample size, breastfeeding time, Ig concentrations, and the techniques used to determine them. To evaluate the evolution of the number of studies of Ig types and subtypes over the years and the evolution of the techniques used for the determinations, all 75 articles were included in the synthesis. However, to evaluate the levels throughout the breastfeeding and to analyze the global proportions, only studies in which the centrality measure was the mean were used. Finally, to create the tables for each Ig type and subtype, as well as for the analysis of Ig levels and global proportions, the studies in which the lactation phase was not specified were excluded.

This review is not a systematic review, since not all the PRISMA items were followed [46]. The search strategy by the electronic databases PubMed and Scopus was described, but we did not restrict the search to randomized clinical trials and the quality of the evidence of the articles was not assessed. Since the first objective of the study was to establish the Ig concentration in breast milk from the literature from the beginning of the Ig studies, we had to include all the articles found, even if they were not randomized clinical trials. 

## 3. Results and Discussion: Immunoglobulins in Breast Milk

### 3.1. Evolution of Studies Quantifying Ig in Breast Milk

**Figure 3:**
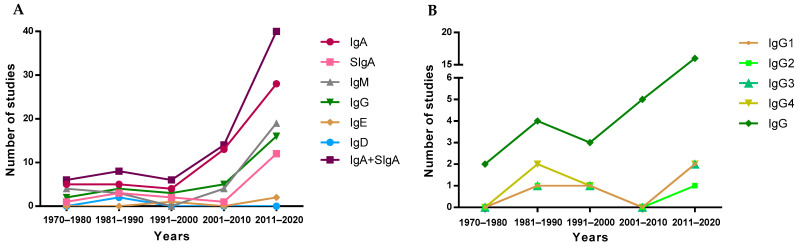
Evolution of the number of studies quantifying Ig types (**A**) and IgG subtypes (**B**) over the years.

To visualize the evolution of the studies over the years, the included articles were separated into periods of time, depending on their publication year, to observe study trends (Figure 3). Overall, since the first studies describing the presence of IgA in breast milk took place in the 1970–1980 period [14,47,48,49,50], a reduced number of studies addressed the quantification of the overall Ig types in the following 30 years. However, a clear increase in the number of articles was found later, specifically those involving the quantification of IgA. This pattern is not followed by IgE or IgD, which are the least studied Igs in breast milk, and only four [51,52,53,54] and two [54,55] articles, respectively, have been found describing the presence of these Igs in breast milk (Figure 3A). It has to be taken into account that although IgA is the most studied Ig type over time and SIgA is the main form of IgA found in breast milk, the majority of articles refer to this Ig type as IgA without specifying whether the IgA quantified was secretory or not. For this reason, in Figure 3A, there is an evolution line for IgA and another for SIgA in addition to the line including both types of IgA. The evolution of the articles describing the IgG subtypes in breast milk was very similar, since they were usually studied together, either in the 80s by ELISA [56] or recently by Luminex assays [41]. 

### 3.2. Techniques to Identify and Quantify Ig in Breast Milk

**Figure 4:**
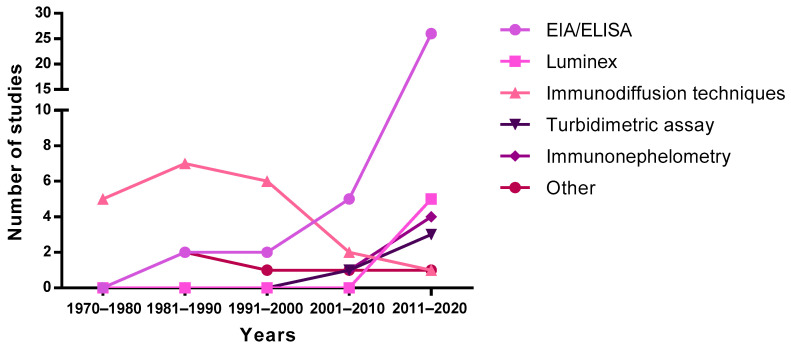
Evolution of the techniques used to measure immunoglobulin levels over the years. EIA, enzyme immunoassay; ELISA, enzyme-linked immunosorbent assay.

The first studies published in the 70s and 80s describing concentrations of Ig in breast milk used immunodiffusion techniques, such as single radial immunodiffusion. Subsequently, this technique was replaced by enzyme-linked immunosorbent assays (ELISA) and bead-based immunoassays (e.g., Luminex), showing an exponential increase from 2000 onwards (Figure 4). It should be taken into account that the quantification of Ig using these methodologies could introduce an almost twofold variation in levels, thus affecting the absolute concentrations described in the literature due to the methodology used [57]. Turbidimetric and immunonephelometric assays have also been used lately in quick routine analysis. In addition, mass spectrometry has also been used lately for all types of milk protein quantification [58]. Overall, ELISA techniques seem to be preferred due to their sensitivity and potential to particularly target SIgA.

### 3.3. Evolution in the Immunoglobulin Profile during the Lactation Period: The Breast Milk Immunoglobulinome

When we study the complete set of metabolites in a cell, tissue, organ, or organism, we refer to them as metabolome; when the attention is focused on the set of expressed proteins, we call it proteome; and if we concentrate our attention on the set of all RNA transcripts, including coding and noncoding, in an individual or a population of cells, we call it transcriptome. Thus, the set of Igs present in a particular fluid or organic compartment could be referred to as immunoglobulinome. Overall, this immunoglobulinome should also be established at a given time and under defined conditions.

In line with this, breast milk is a dynamic fluid whose levels and proportions of Igs change during lactation. This characteristic profile, then, is different at each stage: colostrum, transition, and mature milk. The overall pool of Igs in breast milk includes not only IgA, but also, in lower proportions, the other Ig classes (IgM, IgG, IgE, and IgD), and more recently, the subclasses of IgG have also been studied. Overall, and taking into account the previous considerations, to refer to this particular mixture of Igs at any specific period, in this review we will use the term “breast milk (BM) immunoglobulinome”.

**Figure 5:**
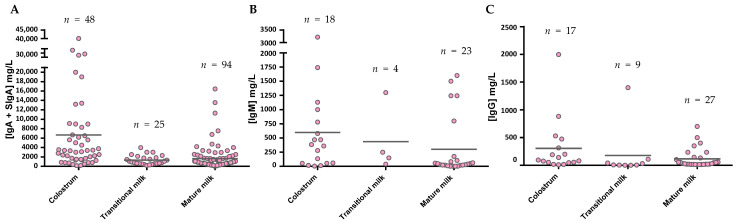
IgA (**A**), IgM (**B**), and IgG (**C**) levels presented in the literature throughout the different phases of breastfeeding. The mean values from each Ig were calculated and shown in the graph using the values provided in the articles for a particular group, independently of the number of samples they are derived from. Figure A takes into account both the determinations obtained from IgA studies and those that claim to measure SIgA specifically.

After considering all the articles published referring to Ig composition in breast milk using the criteria established in the Material and Methods section, the data have been compiled and organized in different tables according to the type of Ig. The tables include the critical aspects determining the Ig concentration described: type of milk (collection day or period), main population characteristics (number of samples analyzed, location, etc.), and finally, the method used for its analysis. All these factors can have an influence in the final concentration described. Studies involving colostrum were considered from d1–d5, the transition period from d6–d15, and from then on mature milk. The mean values from each study, independently of the number of samples they are derived from, have also been compiled and expressed together in Figure 5. 

The levels of IgA (Figure 5A), as described in the individual studies evaluating this concentration in different stages of lactation [59,60,61,62,63,64,65], displayed the highest values in colostrum (~7500 mg/L), with lower levels in transition and mature milk (~1600–2000 mg/L). Due to the variability among studies, no clear differences between transition and mature milk IgA content can be observed. The number of reports studying IgM content in breast milk is much lower than those evaluating IgA, and very few focused on the transition period (Figure 5B). However, a decreasing tendency in IgM content can be observed from colostrum (~600 mg/L) to transition milk (~430 mg/L) and finally to mature milk (~260 mg/L). With regard to IgG, since this is the least studied Ig in breast milk, the results shown here come from a very few studies (Figure 5C), and the overall results are influenced by particular studies with very high values (>800 mg/L). In any case, their levels amount to 180–1100 mg/L. IgE and IgD are minimal in the BM immunoglobulinome at any stage studied, and very few studies have found their presence, as will be further discussed later.

**Figure 6:**
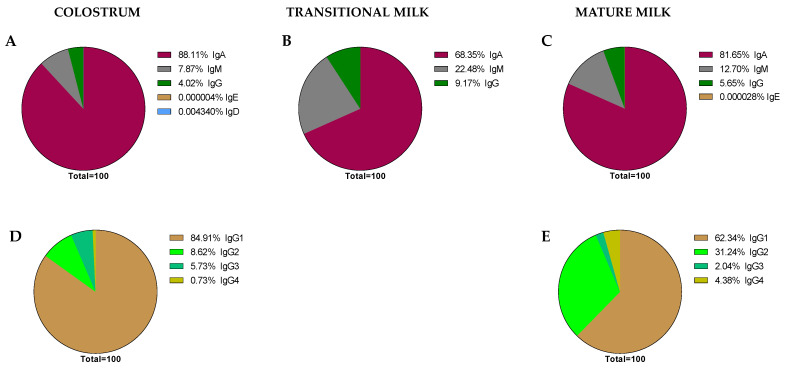
Global proportions from the immunoglobulin concentrations found in the literature. Proportions of Ig classes are expressed in each stage of lactation: colostrum (**A**), transition (**B**), and mature (**C**) milk. IgG subclass proportions were expressed in colostrum (**D**) and mature milk (**E**), as there are no current data for transition milk in this regard.

The great variability in terms of Ig concentration makes it difficult to compile results and draw conclusions; thus, their relative proportion may help to make the data more comparable among studies. However, very few studies reported all three levels of IgA, IgM, and IgG [41,45,52,66,67]. Thus, an overall distribution of Ig proportions was calculated on the basis of the mean values obtained for all values and is shown in Figure 6. 

As expected, it can be observed that IgA is the predominant Ig in the BM immunoglobulinome at any stage of lactation; however, based on our calculations, it seems that the relative proportion of IgA is higher in colostrum (~88.11%) than in transition or mature milk (~68.35–81.65%). It is interesting, though, that the lower proportion in these two last stages of lactation seems to be due to a higher proportion of IgM (~22.45–12.70% vs. ~7.87% in colostrum) in the transition and mature periods. However, these proportions, as noted before, are calculations derived from the current values found in the literature and may not reflect the real BM immunoglobulinome, which can only be derived after having real data from independent studies taking into account all types of Ig in the same sample and at different collection time points.

Aside from some old studies dating mostly from the 80s [56,68], only in the last 10 years, and due to the use of the Luminex techniques, have the studies on the BM immunoglobulinome addressed the IgG subtypes in more depth [41,52]. In this case, the proportion of IgG1, IgG2, IgG3, and IgG4, the main human isotypes [31], have been described in colostrum (Figure 6D) and mature milk (Figure 6E). However, there are no available data on IgG isotypes during the transition period. Regarding their relative proportions, the IgG1 percentage is the highest, followed by IgG2, IgG3, and IgG4. This particular composition, with a predominance of the Th1 response (IgG1 + IgG2 + IgG3) over the Th2 response (IgG4), suggests the breast milk regulatory activity on the neonatal Th1/Th2 balance to minimize the Th2 environment that predominates in the intrauterine space [38,69]. The ratio between these IgG can be of importance in observational studies evaluating the factors influencing breast milk immune composition. A certain diet or particular situations (delivery type or length of gestation period) may lead to changes in this balance that deserve to be studied in depth in the future. 

### 3.4. IgA Concentration in Breast Milk

IgA is the most important class of Ig provided by breast milk to the infant, as it acts in the intestine when the SIgA produced by the infant is still in development. In this sense, IgA is also the most studied Ig in breast milk, being quantified properly, based on our inclusion criteria in 53 of the 75 articles selected. However, when the values obtained are distributed in the three types of milk (colostrum, transition, and mature) or in more specific periods, and the analysis technique and population of study characteristics are also considered, the number of studies for each group are not so many and the variability is high. With regard to the sampling period, the studies describing IgA levels in colostrum (d1–d5) are summarized in Table 1, in the transition period (d6–d15) in Table 2, and in mature milk, in samples after d15, in Table 3. It has to be considered that there exist some results including data in between the above-established periods, thus making the organization of data more difficult.

IgA in colostrum is quantified in samples from the first day of production, considered by some authors as day 0 [45,70,71,72,73] or day 1 [74,75,76,77]. Some studies collected samples on other specific days during this milk period: on day 2 [72,78], on day 3 [45,79,81], on day 4 [41,59,76], or on day 5 [77,81,83]. The rest of the remaining studies have performed the sampling in a certain period of days on just a couple of days [42,60,62], a three-day period [47,66], or even in the whole time in which the breast milk is considered colostrum [64,84], [48]. In some articles, the precise collection time is not specified [14,50,52,85]. The concentration of IgA in this period is quite variable, as commented on in a previous section. Only 11 of the 60 types of samples included (18%) provided values under the 1 g/L, and although some of them are derived from just one mother [34,73] or in particular situations, such as cow’s milk allergy (CMA) or ulcerative colitis (UC) [73,79,85], others involved healthy women [77,81]. Most of the studies show values of between 1 and 10 g/L, specifically in 43 of the 60 populations studied (72%, Table 1). Finally, only six sets of samples (10%) were quantified with values higher than 10 g/L, with some of them reaching concentrations of 30–40 g/L [45,70,71].

Transition milk is the changing fluid established between day 5 and day 15 of lactation. This period, as its name indicates, includes the milk changes in the nutritional composition from colostrum to mature milk, but that also affects the immune components such as the Igs, and especially the IgA. Not many studies on IgA quantification are focused only on this period [61,86,87,88,89,90], and the data compiled also come from studies examining IgA levels in different periods, including this one (Table 2). Overall, 40 sets of transition milk samples from 19 studies quantified IgA. 

In this case, the proportion of the studies describing levels of IgA < 1 g/L or 1–10 g/L are similar: 22/40 (55%) and 18/40 (45%), respectively. The highest value found for healthy donors is ~2.3 g/L corresponding to mothers from Gabon on day 7 [75] and for samples from premature deliveries, which also reached values of ~2–3 g/L [61,76]. Thus, in contrast to the colostrum samples, none of them exceeded the 10 g/L, showing a decreasing IgA concentration pattern in this period.

The sampling period runs between d5 and d15. Some studies quantified IgA on a specific day, either at the beginning, d6 or d7 [45,59,65,75,80], some of them in the middle period, d10 [76], or at the end, d14 or d15 [45,59,76,77,80,90]. Many studies collect samples during a 2–3-day period [60,62,86] or even during longer periods [64,87]. Not all studies expressed the sampling time clearly, and some expressed this using the week as the temporal unit [89,91] or the sampling period was in between colostrum and transition milk or transition milk and mature milk [61].

Finally, quantification of IgA in mature milk is provided in more studies than for colostrum or transition milk (Table 3). In fact, values are found in studies that evaluated the two previous types of milk samples as well, but also in 16 new projects, constituting a total of 109 sets of samples. The sampling period starts in the third week of lactation [59,75,80] and lasts for 24–26 months [92,93,94]. However, the milk obtention is performed on a particular day in early mature milk, such as day 21 [59,75,80], day 28 [59,77,80], or on specific days later on, such as those studies analyzing samples from day 42 [45] or day 56 [80], among others. Besides this, some of the studies included samples from a narrow period of days, e.g., 2–4 days [60,86,95], although most of them collected samples during a longer interval of weeks [88,96,97] or even expressed in certain months [81,88,98]. In addition, some sampling is performed during very long periods, such as for 4–6 months [48,88,92].

With regard to values of IgA described in the selected literature in mature milk, most of the sets of samples analyzed, 70/109 (65%), displayed concentrations of <1 g/L, whereas almost all the rest, 38/70 (35%), comprised between 1 and 10 g/L. There is only one study that describes values >10 g/L corresponding to healthy mothers from the USA [67]. Thus, the overall quantification from colostrum to mature milk shows that the proportion of analyzed milks with a low concentration of IgA (<1 g/L) increases from the first days of lactation (18%) to intermediate samples (55%) up to the end of the period (65%). In contrast, the proportion of milks with the highest levels of IgA (>10 mg/L) was around 10% in colostrum and almost absent in the next two types of milk.

Considering all three periods of lactation, the number of samples included in such sets of samples analyzed is very diverse, with most of them (~60–65%) comprising of 10–60 samples. The rest of the studies included lower numbers of samples (<10 samples, ~30–35%), and only ~5–10% used larger sample sizes (>100 samples). This proportion pattern is maintained independently of the type of milk (colostrum, transition, and mature milk).

Another critical point in establishing IgA concentration in breast milk is differentiating whether IgA is quantified in its dymeric (SIgA) or monomeric form. From the studies included in this review of IgA quantification, only ~40% of them specifically detailed that the values of IgA provided were evaluating SIgA. This proportion is quite consistent in colostrum, transition, and mature milk, whose proportions are 36%, 23%, and 42%, respectively. This does not mean that the rest of the studies are only quantifying monomeric IgA, it is just that in most of these studies, this information is not properly provided. Although many factors influence the concentration of IgA in breast milk, it can be suggested that this aspect could be critical in the enormous IgA variability. 

The technique used for IgA or SIgA quantification could have an influence. Some techniques, such as RIT, seem to have been used some years ago, whereas bead-based immunoassays have been included more recently. However, the ELISA seems to be the preferred technique used. Independently of the technique, the values found vary greatly within the same technique; thus, the wide variability does not seem to be associated with a particular approach. However, it is clear that if SIgA is quantified and specified in the article, the authors mainly use the ELISA technique, because the multitarget approaches, such as the Luminex, do not allow the quantification of this particular form of IgA. 

### 3.5. IgM Concenration in Breast Milk

From the current bibliographic research, only 29 of the 75 selected articles provide data regarding IgM concentrations in breast milk (Table 4). 

In colostrum, available data include IgM levels quantified as early as on the first day of lactation [45,47,48] or on other specific days [45,49,50], although most of the studies comprised samples from either a part (d1–d3 or d2–d4) of the period [42,45,49,62] or even the whole period (d1–d5) of colostrum production [78]. In some articles, the precise collection time is not specified [66].

The concentration of IgM in transition milk is quite variable, due to the intrinsic characteristics of this dynamic period, but also due to the great variability in sample collection times. The sampling ranges from d5, d6, or d7 [65,66] to d14 or d15. However, some of the studies evaluating IgM concentration in transition milk comprise a sampling period [66,75]. The main confusing aspect is when sampling is obtained in a period that starts as transition milk (d5–d15) but continues on to the mature milk stage (>15 d) [52,82,104]. There are even studies detailing the sampling time in weeks [75].

IgM quantification in mature milk depends highly on collection timing, which is even more spread out and usually performed in intervals expressed in months, such as m1–m12 [67], m3–m18 [100], or even longer periods (m3–m26) [105]. In many studies, IgM levels have been described in mature milk, but without defining the specific collection time [91,93,95,99,105,106,107].

The studies evaluating the concentration of IgM in breast milk are diverse, and although some of them only include a very few number of samples (1–10) [65,100], most of the studies comprise a higher number of participants (10–60) [67,93]. Only four studies quantified IgM in approaches involving a robust number of participants (60–90) [42,70,74,88].

As mentioned in Section 3.2, the technique used for the determination could also have an influence on the IgM quantification. Most of the studies evaluating IgM in colostrum used RIT, especially those performed a long time ago [47], and only more recent ones applied ELISA techniques [42,78]. IgM quantification in transition and mature milk also included nephelometry [96], turbidimetry [106], and Luminex [91] in addition to the ones mentioned previously. Although it is difficult to draw any conclusion, the data from the studies included here, especially in mature milk, seem to show that the turbidimetric and nephelometric assays provide much higher concentrations of IgM (10–100 times) than those using other techniques, such as ELISA or multiplex assays (Table 4). In line with the comment for IgA, comparative studies using different techniques for IgM quantification in breast milk may be very helpful in providing knowledge about its real abundance and proportion in the overall breast milk immunoglobulinome.

In addition, although the role of SIgA in enhancing host–microbiota symbiosis and providing infant protection is well known, very little is known about SIgM, and even less in the mammary gland and breast milk compartments [108]. This fact leads us to consider that the local production of SIgM by IgM+ plasma cells (PCs) present in the mammary gland and then released to breast milk has not been properly addressed. Thus, as required for SIgA, SIgM should be analyzed in future studies by using specific techniques that allow its differentiation from the monomeric plasma-filtered IgM.

### 3.6. IgG and IgG Subtypes’ Concentration in Breast Milk

The first quantification study of IgG in human breast milk was published back in 1977 by V. Reddy and collaborators in Indian well- and undernourished women by using RIA [48]. Since then, and due to the low concentration found relatively to IgA or even IgM, very few studies have focused on establishing its levels in human breast milk. Specifically, only 31 studies addressing this issue met the inclusion criteria in this review (Table 5).

As described before for IgA and IgM, the literature on IgG levels in breast milk is distributed in the three collection periods, with nine studies describing its values in colostrum, nine in transition milk, and 13 in mature milk. Of these, only one describes the concentration in the three periods [75].

Although there exist a few of publications regarding IgG levels as early as the first day of lactation [45,70,75], or on other days [45,65,78], colostrum IgG levels are mostly expressed over a period of time of 2–4 days [42,62,66,82,109]. Moreover, one article does not provide the precise collection time within this period [50]. Overall, the levels of IgG in colostrum ranged from 10–2000 mg/L, with a cut-off concentration of 200 mg/L in order to divide the studies with lower and higher values at a 50% proportion each. 

Similarly to IgA and IgM, the values provided from the studies evaluating the IgG concentration in transition milk have higher variability among them than those observed in the colostrum samples. One study found levels of IgG in d5–d10 transition milk from 30 Indian mothers at 0.0055 mg/L [87], whereas another study in 60 mothers from Gabon at d7 found mean levels of IgG of 1400 mg/L [75]. The sampling in this period is performed in a few studies on specific days [45,65,75] or comprising a period lasting 2–6 days [62,87,88]. Some studies are less precise and define their collection time during the second week [91] or as “less than two weeks” [89], or even sampling in a period starting in the transition period (d9) together with the first days (d22) of mature milk [95].

To try to establish a mean value for IgG in mature milk is even more complicated due to the length of this period. The collection of breast milk in the different studies is done at very different time points and intervals, which in turn influences the final results. To date, there are data from the early mature milk period (first month of lactation) [75,88,96,105,109], during the first year [45,48,53,67,88,91,98,100,105], but also from mothers lactating for longer (after the first year of the infant’s life [93,94] or even the second one [94]). The oldest study quantifying IgG in breast milk from mature milk did not detail the collection time [50]. Overall, this sampling diversity, together with other factors, led to a great variability in the IgG levels found in breast milk, with an interval of 13–2000 mg/L. However, it must be taken into account that with the exception of a couple of studies performed in mothers from Zaire [98] and Gabon [75], all the rest of the studies provide an IgG mean value lower than 500 mg/L in all cases.

Regarding the number of samples analyzed in a homogeneous group or condition, colostrum results are derived from studies involving 1 [78] to 77 participants [70], transition milk includes data from 1 [65] to 90 [88], and mature milk from 2 [100] to 90 [88]. Considering all studies, only about 20% of them include a number of participants higher than 50.

As described before for IgA and IgM, different techniques have been used for IgG quantification in breast milk. Studies evaluating colostrum levels mostly use RI and ELISA, and only one used nephelometry [75]. IgG concentration in transition and mature milk is more frequently evaluated by ELISA, and Luminex assays have only been used in more recent studies [67,91]. The wide range of concentration of IgG in breast milk in those periods can also be influenced by this. In this sense, and as observed for IgM, results derived from nephelometry measures seem to provide the highest values (>400 mg/L), followed by Luminex assays (32–96 mg/L), and the lowest ones by ELISA or RI (Table 5).

IgG subtypes have been studied in milk in a low number of studies (Table 6). Specifically, they were quantified in a few approaches in the 1980s–1990s [56,68,109] and more recently in another couple of studies in the last decade [41,52]. The type of milk analyzed is colostrum and mature milk, sometimes within the same study, but information regarding the levels of these IgGs in transition milk is lacking. The older studies used RI/RIA, whereas the more recent ones include the bead-immunoassays, with the ELISA technique being most used overall. With regard to levels of the IgG subtypes, as commented in Section 3.3 and Figure 6, the levels and therefore the relative proportions showed a predominance of IgG1 followed by IgG2, IgG3, and IgG4. The variability of the results found for IgA and IgM is also present here, being more dramatic in colostrum than in mature milk. To date, IgG1 values in colostrum ranged from 2248.4 ± 531.8 mg/L (d2, ELISA) [56] to 37.2 mg/L (d1–d5, RI) [109] (more than 50 times), whereas in mature milk the range is narrower, from 10.36 mg/L (d14–d56, Luminex) [41] to 36.70 mg/L (Luminex) [52] (around 3–4 times) (Table 6). This pattern is similar in the other IgG subtypes, suggesting a normalization of values later in lactation. Further studies focused on the abundance and role of these Igs in breast milk, specifically in transition milk, are required.

### 3.7. IgE in Breast Milk

IgE is the main Ig involved in allergic processes, and it is widely studied in infants’ plasma to deepen the knowledge of tolerance acquisition or its concurrence with the presence of allergic manifestations such as eczema, urticaria, asthma, or rhinoconjunctivitis in susceptible populations. These approaches usually involve a great number of participants, and very large prospective cohorts can be found addressing this aspect, or even the role of certain early life interventions [110]. However, very little information is available regarding IgE concentration in breast milk and therefore its role in these infants.

In this context, precise information about levels of IgE in breast milk is only found in more recent articles in comparison with IgA, IgM, or IgG, and just four articles met our inclusion criteria (Table 7). The first one dates from 1996 and was performed in Swedish atopic and non-atopic mothers [51]. Besides this, only two other studies from 2013 and 2018 also provide quantitative data [52,53]. In fact, data on IgE levels have only been described in colostrum from d2–d4 [51] or without detailing the collection point [52], and in mature milk at 3 or 6 months of lactation [53] or without including the precise sampling time [52]. We have not found any article detailing the IgE levels in human breast milk during the transition period. The pattern of abundance and relative proportion of IgE during lactation is still to be discerned.

Regarding the techniques used for IgE quantification, it is difficult to obtain any clear idea of their influence on obtained values due to the limited number of studies and the differences among them; however, the results derived from both the PRIST and the ELISA [51,53] are ~500 times lower than those described by studies using Luminex assays [52]. Future comparative studies between techniques are required to give a clear answer in this regard.

Unlike other Igs in breast milk, IgE has only been quantified in studies involving a small number of samples, with all the groups studied being composed of N < 12, with the exception of the Swedish approach, which involved 39 participants [51].

### 3.8. IgD in Breast Milk

Although IgD was discovered in 1965 by Rowe and Fahey [111] and its functional significance is still enigmatic, it seems to have similar functions to IgM [112]. 

The presence of IgD in breast milk has only been described in a couple of articles, although IgD antibodies directed to a particular antigen in immunized women have also been described in breast milk [113]. In fact, this last article suggested that IgD may participate in local immune responses of human breast tissues and fluids [113].

The IgD levels described in human breast milk have only been studied in colostrum from Californian participants, with the number of participants being >30 in both approaches [54,55]. 

The lack of studies exploring IgD levels could be linked to its poorly understood role in immunity, but also due to the lack of techniques with enough sensitivity to detect its low presence in milk: levels are <0.5 mg/L, whereas most of the available kits in the market have a limit of detection of 6.25 mg/L.

### 3.9. Factors Influencing Breast Milk Immunoglobulinome

From the current bibliographic research (75 selected articles), several studies have focused on finding an optimal milk treatment to avoid the reduction of the bioactive components [43,49,52,56,66,75,87,88,89,93,113]. On the other hand, much attention has been paid to finding the optimal maternal characteristics and the best environment for a better quality of milk, from studies regarding gestational age to those concerning maternal nutrition, as described below. Overall, the available studies evaluating factors influencing the immunological components of breast milk are mainly focused on IgA but not the other Igs.

Firstly, it is thought that the place of residence could have a high impact on milk Ig levels, and there are a few studies that compare milk Ig composition among different populations [67,84]. Ruiz et al. compared the concentrations of several factors among 10 different locations and showed that the lowest median concentration of IgA corresponds to rural Gambian women (with an interquartile range (IQR) of 159.56–334.94 mg/L), and the highest median concentration of IgA belonged to mothers from Washington (with an IQR of 849.41–2112.45 mg/L) [67]. They also saw a high variability for IgM and IgG levels, but not as high as those for IgA. Another study from 2017 reported that women residing in Burundi had higher levels of IgA than those residing in Italy [84]. However, Igs not only vary from mothers of different regions [67,84], but differences also exist if we compare women of the same community. Surprisingly, the difference between subjects can be 50-fold higher [41,42,43,44,45], and this fact could limit the study of the influence of other factors.

The concentrations of Igs and other bioactive components tend to decrease from colostrum to mature milk [34,60,64,114], as discussed earlier. However, due to the high variability from different sampling approaches and techniques, as we can see in the previous tables, and the variation among subjects, this pattern is not always observed. Nevertheless, some studies have monitored the variations over time, and for instance, one particular study reported that Ig levels followed an inverse U-shaped pattern from month 1 to month 6 of breastfeeding [115]. 

Gestational age is the most studied factor, since it is highly associated with changes in breast milk composition, especially with regards to the IgA levels. The other types of Igs have been less studied based on gestational age, as reflected in the tables above. Human breast milk from mothers of preterm infants increases the IgA concentration to accelerate the development of the immature immune system and provide higher resistance to infections [41,59,60,61,71,76,86,95,99]. Koenig et al., in addition to reporting this increase in IgA, IgG, and total protein in the breast milk of mothers with premature infants, also observed a lower percentage reduction in these components after milk pasteurization, indicating that these immunologic factors may be more resistant in order to be a compensatory protective mechanisms for preterm babies [66].

Previous studies showed controversial results regarding maternal nutrition. Malnourished mothers were reported to have a lower concentration of IgA in colostrum [116,117] and in mature milk at 4 months after delivery [98]. Moreover, Fujita et al. also saw differences in milk SIgA depending on the maternal nutrition but taking into consideration the sex and the age of the neonates, since younger and/or male infants are more susceptible to infections and the milk composition varies depending on the infant’s requirements [118]. Surprisingly, they observed that milk SIgA was lower for younger infants among vitamin A-deficient mothers than for older ones, and that milk SIgA was lower for male infants among mothers with low mid-upper arm circumference [119]. Overall, this may suggest that the reduction in breast milk SIgA due to a state of malnutrition can be accentuated with high infant vulnerability to infection. However, Demers-Mathieu et al. suggested that the free secretory component is more impacted by maternal nutrition than SIgA in breast milk [120]. Indeed, the concentration was higher in women who rarely eat junk food (0–2 times per month) than in mothers who frequently eat junk food (1–4 times per week) [120]. By contrast, other scientists have seen in their studies that the nutritional status reflected by the body mass index does not affect the levels of IgM, IgA, and IgG in human milk [78], but differences could be seen studying the nutritional status from other perspectives. Surprisingly, Urwin et al. reported that mothers who consumed two 150 g portions of salmon per week from week 20 of pregnancy until birth had lower levels of breast milk SIgA than those who rarely ate oily fish [77]. However, the first group also consumed it very rarely before the study. Further studies are needed to clarify this controversy regarding the immunomodulatory effect on breast milk due to the maternal diet. In this regard, diet and maternal microbiota are intimately related, and therefore the influence of the microbiota on the intestine or even on the mammary gland of the lactating mothers might be another key factor influencing Ig pattern that should be explored. To date, no research is available regarding this impact. 

Regarding the development of neonatal tolerance, it has been reported that breast milk IgA is found in lower concentrations in mothers whose infants became allergic to cow’s milk [79,85]. Moreover, maternal diet seems to have an important role, since an avoidance of cow’s milk decreases casein- and beta-lactoglobulin (BLG)-specific IgA in breast milk and induces lower casein-and BLG-specific IgG1 and BLG-specific IgG4 levels in serum in offspring than in those with mothers who consume cow’s milk [121]. The decrease in these specific Igs is related to a high probability of cow’s milk allergy in infants [79,121]. 

Apart from maternal nutrition, other important influences must also be highlighted. It has been strongly established that perinatal maternal stress is associated with prejudicial effects on the infant and the mother, such as the high incidence of depression in the mothers [122] and cognitive and behavioral problems in the infants [123,124]. However, little is known about how this stress could influence the immunological components of human milk. In 2019, an article was published in which it was reported that the postnatal maternal psychological stress was related to a reduction in breast milk SIgA [125], suggesting that infants may not obtain enough immunological protection. In contrast, Aparicio et al. recently reported that neither stress nor milk cortisol were related to the immune components of human milk. However, they considered that it would be interesting to repeat the study with more subjects and with higher levels of psychosocial distress [91]. Other maternal pathologies during pregnancy could also participate in BM immunoglobulinome composition. A reduction in SIgA breast milk was observed in mothers with gestational diabetes mellitus [90] and with inflammatory bowel disease [53].

As we commented before, the infant’s requirements are possible determinants of the milk composition, as IgA seems to be increased in mothers with a preterm delivery. Other evidence is that SIgA and IgG seem to increase in breast milk during any infant and/or maternal infection [104]. However, Riskin et al. only focused on active infection in the nursing infants, and changes were not observed in the breast milk SIgA, but in the white blood cells [126]. It seems that during a maternal and/or infant infection, a strong leukocyte response appears to be accompanied by a variable humoral immune response [104], probably more stable in a maternal infection than in an infant one. Furthermore, maternal vaccination has demonstrated an increase in breast milk-specific antibodies against the respective pathogens [127,128,129,130]. Currently, the vaccines recommended during pregnancy are for tetanus, pertussis, and influenza. Vaccines against other diseases are still being tested to ensure they are safe in pregnancy [131]. With regard to COVID-19 disease, an increase in specific IgG in breast milk has been reported in one woman with COVID-19 [132], and a notable presence of specific IgA in milk has been found in eight recovered women [133]. Further studies should be performed with a higher number of samples to test these findings and to confirm this type of immunity transfer through breastfeeding.

Other factors could interfere in BM immunoglobulinome, but the evidence is not very strong. For instance, breast milk from smokers tends to decrease their SIgA levels [103]. The maternal age, BMI, parity number, and mode of delivery [120] seem not to modify the milk Ig composition [77,78,103].

**Figure 7:**
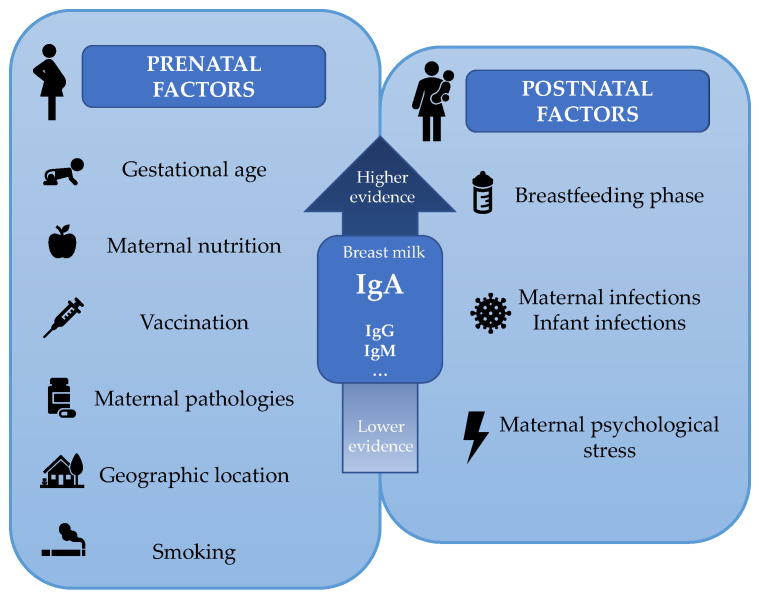
Prenatal and postnatal factors influencing breast milk immunoglobulinome. The factors are displayed from the highest to lowest level of evidence.

In conclusion, several prenatal and postnatal maternal characteristics seem to influence the BM immunoglobulinome composition, especially the IgA concentration (Figure 7). 

Although more research elucidating the effect of maternal factors is required, it is of great interest to find that BM immunoglobulinome changes depending on maternal and infants’ necessities. This knowledge could be used to improve the composition of milk and therefore children’s health through lactation. 

## 4. Conclusions

The current report is an extended review showing the great variability in BM immunoglobulinome composition among studies and the major influences on BM immunoglobulinome changes. The main conclusions drawn from the review are:

- Although IgA is the most studied Ig in breast milk, the other Igs are gaining attention.
- SIgA is the main form of IgA found in breast milk, but several articles are not precise enough in their determination.
- The technique used may have an influence on the outcome. ELISA and bead-based immunoassays (Luminex) are gaining importance and displacing other techniques, although the latter is not used for determining SIgA.
- The sampling period is critical for the quantification of Igs.
- There is a low number of studies addressing other Ig types in breast milk, both in the characterization of the BM immunoglobulinome and in the study of the influence of maternal factors, especially in the transition phase.

Future studies, with precise and detailed sampling procedures, evaluating levels and proportions of Igs over time in an adequate number of samples and with detailed methodology, are required to establish the real importance of BM immunoglobulinome and its influencing factors.

## Figures and Tables

**Table 1:** IgA in Colostrum.

Breastfeeding Phase and Time	Study	Year	Population Characteristics N, Location, Particular Characteristics (Age)	Measure of Centrality and Spread	Concentration and Distribution (mg/L)	Analysis Method
**Colostrum**						
h24	[70]	2015	77, Brazil, Healthy	Mean (SD)	28,502 (25,672)	ELISA
d0	[71]	2006	14, Turin, Term delivery	~Mean	20,000	#
d0	[71]	2006	16, Turin, Preterm delivery	~Mean	40000	#
d0	[45]	1982	11, New Zealand, Healthy	Mean (Range)	32,000 (1500–83,700)	RI **
d0	[72]	2012	44, Brazil, Healthy	Mean ± SD	8291 ± 3376	T **
d0	[73]	2011	1, Bulgaria, Healthy (34)	Value	137.4	EIA **
d0	[73]	2011	1, Bulgaria, Mother with UC (29)	Value	408.5	EIA **
d1	[74]	2018	90, Turkey, Healthy	Mean ± SD	29,370 ± 15,000	N
d1	[75]	2011	60, Gabon, Healthy	Mean ± SEM	13,400 ± 5900	N
d1	[76]	2005	10, Brazil, Term delivery	Median (Range)	28,310 (11,900–41,400)	IDQR **
d1	[76]	2005	10, Brazil, Preterm delivery	Median (Range)	213,890 (88,550–468,080)	IDQR **
d1	[77]	2012	9, Southampton, Control group	Median (25th–75th)	3130 (1760–7040)	ELISA **
d1	[77]	2012	9, Southampton, Salmon supplementation	Median (25th–75th)	1130 (770–3240)	ELISA **
d0–d3	[47]	1972	133, Guatemala	Mean	3330	RI
d1–d2	[62]	1985	20, Moscow, Healthy (17–41)	Mean (SD)	6190 (1100)	SRI
d2	[78]	2006	31, Healthy, Bangladesh	Mean ± SD	5630 ± 1640	ELISA
d2	[72]	2012	44, Brazil, Healthy, Non-supplemented	Mean ± SD	3439 ± 1772	T **
d2	[72]	2012	52, Brazil, Healthy, Vit A supplementation	Mean ± SD	5012 ± 545	T **
d3	[79]	2000	48, Helsinki, infants with CMA	Mean (95%CI)	380 (240–280)	RI
d3	[79]	2000	39, Helsinki, infants without CMA	Mean (95%CI)	820 (990–1510)	RI
d3	[65]	2009	1, Brazil, mother with CVID	Value	26	ELISA
d3	[65]	2009	1, Brazil, mother with CVID	Value	0.7	ELISA
d3	[45]	1982	11, New Zealand, Healthy	Mean (Range)	9000 (630–32,800)	RI **
d3	[80]	2013	41, Tokyo, Healthy, Primipara	Mean	2241	EIA **
d3	[81]	1982	7, Ethiopia, Underprivileged	Mean ± SD	1690 ± 480	ELISA **
d3	[81]	1982	5, Ethiopia, Privileged	Mean ± SD	5600 ± 6540	ELISA **
d3 ± d1	[60]	2011	22, Spain, Term delivery	~Mean	6500	ELISA
d3 ± d1	[60]	2011	10, Spain, Preterm delivery	~Mean	9100	ELISA
d3 ± d1	[60]	2011	10, Spain, Very preterm delivery	~Mean	2500	ELISA
d2–d3	[42]	2004	82, Brazil, Healthy (21–41)	Median (range)	7500 (920–55,000)	ELISA
d4	[59]	1981	8, Durham, Preterm delivery	~Mean	4500	RI
d4	[59]	1981	5, Durham, Term delivery	~Mean	3400	RI
d4	[76]	2005	10, Brazil, Term delivery	Median (Range)	1290 (680–1790)	IDQR **
d4	[76]	2005	10, Brazil, Preterm delivery	Median (Range)	8130 (4730–118,890)	IDQR **
h96	[41]	2015	15, Spain, Preterm delivery	Median (IQR)	8980 (560–17,400)	Luminex
d1–d4	[82]	2013	11, Portugal, Healthy, Unprocessed milk	Mean ± SD	1728 ± 34	ELISA
d2–d4	[66]	2005	36, Brazil, <32 w of g.a., Non-pasteurized milk	Mean ± SD	3102 ± 1360	RI
d2–d4	[66]	2005	36, Brazil, <32 w of g.a., Pasteurized milk	Mean ± SD	2032 ± 1115	RI
d2–d4	[66]	2005	32, Brazil, 32–36 w of g.a., Non-pasteurized milk	Mean ± SD	3004 ± 1303	RI
d2–d4	[66]	2005	32, Brazil, 32–36 w of g.a., Pasteurized milk	Mean ± SD	1331 ± 0878	RI
d2–d4	[66]	2005	33, Brazil, >37 w of g.a., Non-pasteurized milk	Mean ± SD	2250 ± 1267	RI
d2–d4	[66]	2005	33, Brazil, >37 w of g.a., Pasteurized milk	Mean ± SD	858 ± 521	RI
d3–d4	[62]	1985	20, Moscow, Healthy (17–41)	Mean (SD)	2390 (558)	SRI
d1-d5	[64]	2001	42	Mean (SEM)	19,020 (3110)	IN
d5	[83]	2011	Helsinki, Non-atopic mothers	Mean (SD)	1367 (1062)	SRI
d5	[83]	2011	Helsinki, Atopic mothers	Mean (SD)	1252 (1090)	SRI
d5	[81]	1982	7, Ethiopia, Underprivileged	Mean ± SD	720 ± 270	ELISA **
d5	[81]	1982	5, Ethiopia, Privileged	Mean ± SD	790 ± 330	ELISA **
d5	[77]	2012	26, Southampton, Control group	Median (25th–75th)	690 (510–1070)	ELISA **
d5	[77]	2012	28, Southampton, Salmon supplementation	Median (25th–75th)	550 (410–680)	ELISA **
d1–d5	[48]	1977	17, India, Well-nourished women	Mean ± SEM	3359 ± 373.9	RI
d1–d5	[48]	1977	10, India, Under-nourished women	Mean ± SEM	3743 ± 421.3	RI
d1–d6	[84]	2018	22, Burundi, Healthy (24.30)	Mean (IQR)	2780 (1450–22,200)	IT
d1–d6	[84]	2018	48, Italy, Healthy (37.39)	Mean (IQR)	1480 (890–2670)	IT
*	[52]	2013	10, Spain, Healthy, Untreated milk	Median (IQR)	7180 (6530–7640)	Luminex
*	[52]	2013	7, Spain, Healthy, Pasteurized milk	Median (IQR)	3620 (2450–4780)	Luminex
*	[14]	1971	15, Oslo, Healthy	Mean	13,180	SRI
*	[50]	1978	24, Caucasian and Turkish women, Healthy (16–40)	Mean ± SD	3542 ± 992	RI
*	[85]	1991	102, Helsinki, Healthy infants	~Mean	1500	SRI **
*	[85]	1991	7, Helsinki, CMA infants	~Mean	500	SRI **

SD, standard deviation of the mean; SEM, standard error mean; IQR, interquartile range; EIA, enzyme immunoassay; ELISA, enzyme-linked immunosorbent assay; IDQR, quantitative radial immunoassay; #, SDS-PAGE and immunoblotting; N, nephelometry; T, turbidimetry; IN, immunonephelometry; IT, immunoturbidity; RI, radial immunodiffusion; SRI, single radial immunodiffusion; CMA, cow’s milk allergy; CVID, common variable immunodeficiency; CD, Crohn disease; UC, ulcerative colitis; g.a., gestational age; * Data are not specified. ** The analytical method indicates that SIgA is quantified.

**Table 2:** IgA in transition milk.

Breastfeeding Phase and Time	Study	Year	Population Characteristics N, Location, Particular Characteristics (Age)	Measure of Centrality and Spread	Concentration and Distribution (mg/L)	Analysis Method
**Transition milk**						
d5–d6	[62]	1985	20, Moscow, Healthy (17–41)	Mean (SD)	782 (312)	SRI
d6	[45]	1982	11, New Zealand, Healthy	Mean (Range)	1450 (400–3140)	RI **
d7	[65]	2009	1, Brazil, mother with CVID	Value	0.9	ELISA
d7	[65]	2009	1, Brazil, mother with CVID	Value	0.7	ELISA
d7	[75]	2011	60, Gabon, Healthy	Mean ± SEM	2300 ± 2000	N
d7	[59]	1981	10, Durham, Preterm delivery	~Mean	3000	RI
d7	[59]	1981	8, Durham, Term delivery	~Mean	1500	RI
d7	[80]	2013	41, Tokyo, Healthy, Primipara	Mean	2241	EIA **
d6–d8	[86]	2011	5, USA, Term delivery	~Mean	600	ELISA **
d6–d8	[86]	2011	15, USA, Preterm delivery	~Mean	690	ELISA **
d7–d8	[62]	1985	20, Moscow, Healthy (17–41)	Mean (SD)	575 (139)	SRI
d10	[76]	2005	10, Brazil, Term delivery	Median (Range)	890 (630–1140)	IDQR **
d10	[76]	2005	10, Brazil, Preterm delivery	Median (Range)	5455 (2000–17,640)	IDQR **
d10 ± d2	[60]	2011	22, Spain, Term delivery	~Mean	800	ELISA
d10 ± d2	[60]	2011	10, Spain, Preterm delivery	~Mean	1100	ELISA
d10 ± d2	[60]	2011	10, Spain, Very preterm delivery	~Mean	900	ELISA
d0–d10	[61]	1984	18, Finland, Preterm delivery	Mean ± SEM	2510 ± 148	RI
d0–d10	[61]	1984	15, Finland, Term delivery	Mean ± SEM	2128 ± 199	RI
d5–d10	[87]	2018	30, India, Term delivery, Pre-pasteurization	Mean (SD)	0.623 (0.084)	ELISA
d5–d10	[87]	2018	30, India, Term delivery, Post-pasteurization	Mean (SD)	0.436 (0.058)	ELISA
d5–d11	[88]	2016	90, China, Healthy urban mothers	Median (IQR)	1148 (1022)	ELISA
d6–d14	[64]	2001	18	Mean (SEM)	3970 (1450)	IN
Less w2	[89]	1992	16, Canada, Non-ultrasonic homogenized milk	Mean ± SD	414 ± 344	I
Less w2	[89]	1992	16, Canada, Ultrasonic homogenized milk (<45°)	Mean ± SD	367 ± 350	I
Less w2	[89]	1992	16, Canada, Ultrasonic homogenized milk (>55°)	Mean ± SD	186 ± 205	I
d14	[59]	1981	11, Durham, Preterm delivery	~Mean	3000	RI
d14	[59]	1981	8, Durham, Term delivery	~Mean	1100	RI
d14	[45]	1982	11, New Zealand, Healthy	Mean (Range)	750 (500–1100)	RI **
d14	[90]	2013	8, California, Mothers with GDM	~Mean	300	## **
d14	[90]	2013	16, California, Mothers with GDM	~Mean	540	## **
d14	[80]	2013	40, Tokyo, Healthy, Primipara	Mean	1772.8	EIA **
d14	[77]	2012	28, Southampton, Control group	Median (25th–75th)	520 (330–630)	ELISA **
d14	[77]	2012	27, Southampton, Salmon supplementation	Median (25th–75th)	390 (270–510)	ELISA **
w2	[91]	2020	51, Netherlands, Healthy, Term delivery	Median (IQR)	1680 (1080–2090)	Luminex
d13–d15	[86]	2011	5, USA, Term delivery	~Mean	600	ELISA **
d13–d15	[86]	2011	15, USA, Preterm delivery	~Mean	640	ELISA **
d15	[76]	2005	10, Brazil, Term delivery	Median (Range)	755 (530–1300)	IDQR **
d15	[76]	2005	10, Brazil, Preterm delivery	Median (Range)	5765 (1430–20,650)	IDQR **
d10–d20	[61]	1984	12, Finland, Preterm delivery	Mean ± SEM	2552 ± 153	RI
d10–d20	[61]	1984	8, Finland, Term delivery	Mean ± SEM	1740 ± 173	RI

SD, standard deviation of the mean; SEM, standard error mean; IQR, interquartile range; EIA, enzyme immunoassay; ELISA, enzyme-linked immunosorbent assay; IDQR, quantitative radial immunoassay; ##, bradford assay after SIgA purification; ELISA, enzyme-linked immunosorbent assay; N, nephelometry; IN, immunonephelometry; RI, radial immunodiffusion; I, immunodiffusion; SRI, single radial immunodiffusion; IDQR, quantitative radial immunoassay; CMA, cow’s milk allergy; CVID, common variable immunodeficiency; CD, Crohn disease; UC, ulcerative colitis; GDM, gestational diabetes mellitus. ** The analytical method indicates that SIgA is quantified.

**Table 3:** IgA in mature milk.

Breastfeeding Phase and Time	Study	Year	Population Characteristics N, Location, Particular Characteristics (Age)	Measure of Centrality and Spread	Concentration and Distribution (mg/L)	Analysis Method
**Mature milk**						
d21	[75]	2011	60, Gabon, Healthy	Mean ± SEM	4000 ± 2300	N
d8–d22	[95]	2019	36, Oregon, Preterm delivery	~Mean	750	ELISA
d8–d22	[95]	2019	36, Oregon, Term delivery	~Mean	600	ELISA **
d21	[59]	1981	10, Durham, Preterm delivery	~Mean	2400	RI
d21	[59]	1981	7, Durham, Term delivery	~Mean	900	RI
d21	[80]	2013	40, Tokyo, Healthy, Primipara	Mean	1673.8	EIA **
d20–d22	[86]	2011	5, USA, Term delivery	~Mean	600	ELISA **
d20–d22	[86]	2011	15, USA, Preterm delivery	~Mean	610	ELISA **
d28	[59]	1981	10, Durham, Preterm delivery	~Mean	2900	RI
d28	[59]	1981	5, Durham, Term delivery	~Mean	1000	RI
d28	[80]	2013	40, Tokyo, Healthy, Primipara	Mean	1285.5	EIA **
d28	[77]	2012	23, Southampton, Control group	Median (25th–75th)	380 (310–530)	ELISA **
d28	[77]	2012	23, Southampton, Salmon supplementation	Median (25th–75th)	310 (220–430)	ELISA **
d12–d30	[88]	2016	90, China, Healthy urban mothers	Median (IQR)	6150 (4940)	ELISA
d15–d30	[96]	2017	4, Mexico, Healthy, Unprocessed milk	~Mean	1400	N
d15–d30	[96]	2017	4, Mexico, Healthy, Pasteurized milk (85°)	~Mean	800	N
d30 ± d2	[60]	2011	22, Spain, Term delivery	~Mean	500	ELISA
d30 ± d2	[60]	2011	10, Spain, Preterm delivery	~Mean	500	ELISA
d30 ± d2	[60]	2011	10, Spain, Very preterm delivery	~Mean	500	ELISA
d27–d29	[86]	2011	5, USA, Term delivery	~Mean	600	ELISA **
d27–d29	[86]	2011	15, USA, Preterm delivery	~Mean	650	ELISA **
d20–d30	[61]	1984	8, Finland, Preterm delivery	Mean ± SEM	2518 ± 145	RI
d20–d30	[61]	1984	11, Finland, Term delivery	Mean ± SEM	1716 ± 128	RI
d2–d47	[99]	2018	15, California, Preterm delivery	~Mean	600	ELISA **
d2–d47	[99]	2018	8, California, Term delivery	~Mean	500	ELISA **
d14–d150	[67]	2017	41, Spain, Healthy (>18)	Mean (IQR)	4188.3 (2567.8–5392.4)	Luminex
d14–d150	[67]	2017	40, Ethiopia, Healthy (>18)	Mean (IQR)	3232.2 (2233.7–4695.2)	Luminex
d14–d150	[67]	2017	41, USA/Washington, Healthy (>18)	Mean (IQR)	13,556.0 (8494.1–21124.5)	Luminex
d14–d56	[41]	2015	15, Spain, Preterm delivery	Median (IQR)	6800 (−6300 to 39,900)	Luminex
d15–d75	[64]	2001	21	Mean (SEM)	11,300 (1900)	IN
m1	[81]	1982	7, Ethiopia, Underprivileged	Mean ± SD	430 ± 180	ELISA **
m1	[81]	1982	3, Ethiopia, Privileged	Mean ± SD	610 ± 700	ELISA **
m1	[81]	1982	15, Sweden, Healthy	Mean ± SD	830 ± 310	ELISA **
m1	[81]	1982	10, Guatemala, Rural women	Mean ± SD	630 ± 210	ELISA **
m1	[81]	1982	10, Guatemala, Urban poor women	Mean ± SD	840 ± 550	ELISA **
m1	[81]	1982	10, Guatemala, Urban privileged	Mean ± SD	1020 ± 650	ELISA **
m1	[98]	1995	14, Zaire, Well–nourished women	mean ± SD	3360 ± 1690	RI **
m1	[98]	1995	17, Zaire, Malnourished women	mean ± SD	4720 ± 5000	RI **
d42	[45]	1982	11, New Zealand, Healthy	Mean (Range)	830 (450–1500)	RI **
d56	[80]	2013	19, Tokyo, Healthy, Primipara	Mean	1084.7	EIA **
m1–m2	[88]	2016	90, China, Healthy urban mothers	Median (IQR)	553 (232)	ELISA
m2	[83]	2011	Helsinki, Non-atopic mothers	Mean (SD)	344 (208)	SRI
m2	[83]	2011	Helsinki, Atopic mothers	Mean (SD)	324 (192)	SRI
m2	[85]	1991	100, Helsinki, Healthy infants	~Mean	400	SRI **
m2	[85]	1991	7, Helsinki, CMA infants	~Mean	250	SRI **
w6	[91]	2020	51, Netherlands, Healthy, Term delivery	Median (IQR)	1680 (1080–2090)	Luminex
d70	[45]	1982	11, New Zealand, Healthy	Mean (Range)	710 (350–1150)	RI **
d84	[80]	2013	19, Tokyo, Healthy, Primipara	Mean	973.7	EIA **
w8–w9	[63]	1998	65, Gambia, Healthy	Median (IQR)	625 (376–959)	ELISA
m2–m4	[88]	2016	90, China, Healthy urban mothers	Median (IQR)	557 (312)	ELISA
m4–m8	[88]	2016	90, China, Healthy urban mothers	Median (IQR)	564 (337)	ELISA
m3	[53]	2018	7, Alberta, Healthy	~Mean	250	ELISA
m3	[53]	2018	5, Alberta, Mothers with CD	~Mean	100	ELISA
m3	[53]	2018	11, Alberta, Mothers with UC	~Mean	50	ELISA
m3	[81]	1982	15, Sweden, Healthy	Mean ± SD	510 ± 180	ELISA **
m3	[81]	1982	9, Guatemala, Rural women	Mean ± SD	410 ± 130	ELISA **
m3	[81]	1982	11, Guatemala, Urban poor women	Mean ± SD	600 ± 210	ELISA **
m3	[81]	1982	10, Guatemala, Urban privileged	Mean ± SD	580 ± 240	ELISA **
w12	[91]	2020	51, Netherlands, Healthy, Term delivery	Median (IQR)	1260 (830–1680)	Luminex
w16–w17	[63]	1998	65, Gambia, Healthy	Median (IQR)	666 (399–1125)	ELISA
m4	[98]	1995	14, Zaire, Well-nourished women	mean ± SD	2240 ± 520	RI **
m4	[98]	1995	17, Zaire, Malnourished women	mean ± SD	1670 ± 600	RI **
m6	[81]	1982	16, Sweden, Healthy	Mean ± SD	770 ± 940	ELISA **
m6	[81]	1982	10, Guatemala, Rural women	Mean ± SD	400 ± 220	ELISA **
m6	[85]	1991	65, Helsinki, Healthy infants	~Mean	250	SRI **
m6	[85]	1991	7, Helsinki, CMA infants	~Mean	200	SRI**
m1–m6	[92]	2020	43, Thailand, Healthy (18–40)	Mean ± SD	1108.2 ± 140.6	ELISA
m1–m6	[48]	1977	12, India, Well-nourished women	Mean ± SEM	1196 ± 785	RI
m1–m6	[48]	1977	10, India, Under-nourished women	Mean ± SEM	1181 ± 162	RI
m6	[83]	2011	Helsinki, Non-atopic mothers	Mean (SD)	208 (73)	SRI
m6	[83]	2011	Helsinki, Atopic mothers	Mean (SD)	205 (078)	SRI
m6	[53]	2018	7, Alberta, Healthy	~Mean	350	ELISA
m6	[53]	2018	6, Alberta, Mothers with CD	~Mean	50	ELISA
m6	[53]	2018	7, Alberta, Mothers with UC	~Mean	50	ELISA
w27–w28	[63]	1998	65, Gambia, Healthy	Median (IQR)	680 (451–1008)	ELISA
w39–w40	[63]	1998	65, Gambia, Healthy	Median (IQR)	715 (359–1063)	ELISA
m4–m8	[100]	2016	2, Canada, Milk before spray drying	Mean ± SD	215.80 ± 6.84	ELISA
m4–m8	[100]	2016	2, Canada, Milk before freeze drying	Mean ± SD	262.68 ± 56.40	ELISA
m9	[81]	1982	16, Sweden, Healthy	Mean ± SD	900 ± 730	ELISA **
m9	[81]	1982	10, Guatemala, Rural women	Mean ± SD	430 ± 150	ELISA **
m9	[85]	1991	39, Helsinki, Healthy infants	~Mean	300	SRI **
m9	[85]	1991	7, Helsinki, CMA infants	~Mean	200	SRI **
m1–m12	[93]	2020	26, Wroclaw, Healthy	Mean ± SD	2120 ± 620	ELISA **
m6–m12	[92]	2020	47, Thailand, Healthy (18–40)	Mean ± SD	1295.9 ± 166.7	ELISA
w17–w52	[47]	1972	133, Guatemala	Mean	500	RI
w51–w52	[63]	1998	65, Gambia, Healthy	Median (IQR)	746 (408–1067)	ELISA
>w53	[47]	1972	133, Guatemala	Mean	2420	RI
m10–m15	[101]	1983	269, Bangladesh, Peri-urban mothers with GI	Range	10–1700	ELISA **
m12–m18	[92]	2020	50, Thailand, Healthy (18–40)	Mean ± SD	1242.9 ± 108.0	ELISA
m13–m18	[93]	2020	35, Wroclaw, Healthy	Mean ± SD	2950 ± 1300	ELISA **
m19–m24	[93]	2020	32, Wroclaw, Healthy	Mean ± SD	3350 ± 2220	ELISA **
>m24	[93]	2020	23, Wroclaw, Healthy	Mean ± SD	7550 ± 7160	ELISA **
m18–m24	[92]	2020	44, Thailand, Healthy (18–40)	Mean ± SD	1271.6 ± 145.9	ELISA
m3–m26	[94]	2013	5, Spain, Healthy, Unprocessed milk	Mean ± SD	433.9 ± 17.6	ELISA
m3–m26	[94]	2013	6, Spain, Healthy, Unprocessed milk	Mean ± SD	1646.0 ± 153.4	ELISA
d1–d249	[44]	1994	64, Sri Lanka, (25)	Median (Range)	2340 (300–19100)	ELISA
d1–d205	[44]	1994	29, Asia, Immigrant women (26)	Median (Range)	3100 (200–16400)	ELISA
d1–d310	[44]	1994	75, UK, White women (29.5)	Median (Range)	2500 (200–18200)	ELISA
*	[52]	2013	8, Spain, Healthy, Untreated milk	Median (IQR)	5960 (2810–6790)	Luminex
*	[52]	2013	8, Spain, Healthy, Pasteurized milk	Median (IQR)	2220 (1280–3430)	Luminex
*	[49]	1977	>25, Cardiff, Untreated milk	Mean	50	RI
*	[49]	1977	>25, Cardiff, Heat-treated milk (56° 30 min)	Mean	480	RI
*	[49]	1977	>25, Cardiff, Heat-treated milk (62.5° 30 min)	Mean	390	RI
*	[49]	1977	>25, Cardiff, Heat-treated milk (70° 15 min)	Mean	240	RI
*	[49]	1977	>25, Cardiff, Heat-treated milk (80° 15 min)	Mean	100	RI
*	[50]	1978	19, Caucasian and Turkish women, Healthy (16–40)	Mean ± SD	2163 ± 797	RI
*	[102]	2013	9, Poland, Atopic mothers	Median (IQR)	476.836 (209.2–678.53)	ELISA **
*	[102]	2013	61, Poland, Non-atopic mothers	Median (IQR)	782.47 (614.04–916.69)	ELISA **
*	[103]	2012	40, Beirut, Non-smokers	Mean ± SD	1070 ± 260	IN *
*	[103]	2012	23, Beirut, Smokers	Mean ± SD	780 ± 320	IN *

SD, standard deviation of the mean; SEM, standard error mean; IQR, interquartile range; EIA, enzyme immunoassay; ELISA, enzyme-linked immunosorbent assay; N, nephelometry; IN, immunonephelometry; RI, radial immunodiffusion; SRI, single radial immunodiffusion; CMA, cow’s milk allergy; CVID, common variable immunodeficiency; CD, Crohn disease; UC, ulcerative colitis, GI, giardia infection. * Data are not specified. ** The analytical method indicates that SIgA is quantified.

**Table 4:** IgM in milk.

Breastfeeding Phase and Time	Study	Year	Population Characteristics N, Location, Particular Characteristics (Age)	Measure of Centrality and Spread	Concentration and Distribution (mg/L)	Analysis Method
**Colostrum**						
d0	[45]	1982	11, New Zealand, Healthy	Mean (range)	1130 (230–1700)	RI
h24	[70]	2015	77, Brazil, Healthy	Mean (SD)	3218 (883)	ELISA
d1	[74]	2018	90, Turkey, Healthy, Vaginal delivery	Mean ± SD	1740 ± 1200	N
d1	[75]	2011	60, Gabon, Health, Term delivery	Mean ± SEM	1000 ± 1600	N
d1–d2	[62]	1985	20, Moscow, Healthy (25.5)	Mean (SD)	383 (78)	SRI
d2	[78]	2006	31, Bangladesh, Healthy (18–35)	Mean ± SD	470 ± 90	ELISA
d0–d3	[47]	1972	34, Guatemala	Mean	360	RI
d3	[65]	2009	1, Brazil, mother with CVID	Value	500	ELISA
d3	[65]	2009	1, Brazil, mother with CVID	Value	5.1	ELISA
d3	[45]	1982	11, New Zealand, Healthy	Mean (range)	580 (80–1560)	RI
d2–d3	[42]	2004	82, Brazil, Healthy (21–41)	Median (range)	1125.0 (110.0–31,840.0)	ELISA
d4	[41]	2015	15, Spain, Preterm delivery	Mean (IQR)	780 (−200 to 1770)	Luminex
d1–d4	[104]	2013	5, Australia, Healthy	Range	16.2–56.1	ELISA
d1–d4	[82]	2013	11, Portugal, Healthy, Unprocessed milk	Mean ± SD	280 ± 11	ELISA
d2–d4	[66]	2005	36, Brazil, <32 w of g.a., Non-pasteurized milk	Mean ± SD	17 ± 38	RI
d2–d4	[66]	2005	36, Brazil, <32 w of g.a., Pasteurized milk	Mean ± SD	0.0 ± 0.0	RI
d2–d4	[66]	2005	32, Brazil, 32–36 w of g.a., Non-pasteurized milk	Mean ± SD	5 ± 15	RI
d2–d4	[66]	2005	32, Brazil, 32–36 w of g.a., Pasteurized milk	Mean ± SD	0.0 ± 0.0	RI
d2–d4	[66]	2005	33, Brazil, >37 w of g.a., Non-pasteurized milk	Mean ± SD	14 ± 34	RI
d2–d4	[66]	2005	33, Brazil, >37 w of g.a., Pasteurized milk	Mean ± SD	0.0 ± 0.0	RI
d3–d4	[62]	1985	20, Moscow, Healthy (25.5)	Mean (SD)	53 (16)	SRI
d1–d5	[48]	1977	17, India, Well-nourished women	Mean ± SEM	59 ± 15.8	RI
d1–d5	[48]	1977	10, India, Under-nourished women	Mean ± SEM	53 ± 23.0	RI
*	[52]	2013	10, Spain, Healthy, Untreated milk	Median (IQR)	93.94 (38.79–201.30)	Luminex
*	[52]	2013	10, Spain, Healthy, Pasteurized milk	Median (IQR)	59.36 (14.95–173.92)	Luminex
*	[50]	1978	24, Caucasian and Turkish women, Healthy (16–40)	Mean ± SD	4047 ± 1170	RI
**Transition milk**						
d5–d6	[62]	1895	20, Moscow, Healthy (25.5)	Mean (SD)	135 (40)	SRI
d6	[45]	1982	11, New Zealand, Healthy	Mean (range)	250 (30–1050)	RI
d7	[75]	2011	60, Gabon, Health, Term delivery	Mean ± SEM	1300 ± 800	N
d7	[65]	2009	1, Brazil, mother with CVID	Value	13.7	ELISA
d7	[65]	2009	1, Brazil, mother with CVID	Value	91	ELISA
d7–d8	[62]	1895	20, Moscow, Healthy (25.5)	Mean (SD)	39 (21)	SRI
d5–d11	[88]	2016	90, China, Healthy urban mothers	Median (IQR)	117 (168)	ELISA
d14	[45]	1982	11, New Zealand, Healthy	Mean (range)	150 (30–800)	RI
w2	[91]	2020	51, Netherlands, Healthy, Term delivery	Median (IQR)	64.73 (47.84–97.12)	Luminex
d5–d21	[104]	2013	5, Australia, Healthy	Range	8.2–29.8	ELISA
d5–d21	[104]	2013	1, Australia, Maternal infection	Value	10.2	ELISA
d8–d22	[95]	2019	36, Oregon, Preterm delivery	~Mean	175	ELISA
**Mature milk**						
d21	[75]	2011	60, Gabon, Health, Term delivery	Mean ± SEM	1500 ± 1700	N
d15–d30	[96]	2017	4, Mexico, Healthy (30), Unprocessed milk	~Mean	1600	N
d15–d30	[96]	2017	4, Mexico, Healthy (30), Pasteurized milk (85°)	~Mean	800	N
d12–d30	[88]	2016	90, China, Healthy urban mothers	Median (IQR)	47 (47)	ELISA
m1	[105]	2018	36, Jordan, Healthy, Term delivery	Mean (SD)	103 (31.0)	ELISA
m1–m2	[88]	2016	90, China, Healthy urban mothers	Median (IQR)	35 (31)	ELISA
w6	[91]	2020	51, Netherlands, Healthy, Term delivery	Median (IQR)	38.19 (21.73–61.92)	Luminex
d42	[45]	1982	11, New Zealand, Healthy	Mean (range)	50 (10–160)	RI
d14–d56	[41]	2015	15, Spain, Preterm delivery	Mean (IQR)	21,550 (870–42,220)	Luminex
d70	[45]	1982	11, New Zealand, Healthy	Mean (range)	30 (0–120)	RI
d21–m3	[104]	2013	2, Australia, Healthy	Range	10.6–14.9	ELISA
d21–m3	[104]	2013	7, Australia, Maternal infection	Range	4.5–19.8	ELISA
m4	[105]	2018	36, Jordan, Healthy, Term delivery	Mean (SD)	64 (25.7)	ELISA
m2–m4	[88]	2016	90, China, Healthy urban mothers	Median (IQR)	35 (29)	ELISA
d14–d150	[67]	2017	41, Spain, Healthy (>18)	Mean (IQR)	38.80 (19.92–62.45)	Luminex
d14–d150	[67]	2017	40, Ethiopia, Healthy (>18)	Mean (IQR)	83.93 (45.36–120.48)	Luminex
d14–d150	[67]	2017	41, USA/Washington, Healthy (>18)	Mean (IQR)	18.95 (7.78–36.60)	Luminex
m1–m6	[48]	1977	12, India, Well-nourished women	Mean ± SEM	29 ± 9.2	RI
m1–m6	[48]	1977	10, India, Under-nourished women	Mean ± SEM	58 ± 34.1	RI
m6	[105]	2018	36, Jordan, Healthy, Term delivery	Mean (SD)	48 (18.1)	ELISA
m4–m8	[88]	2016	90, China, Healthy urban mothers	Median (IQR)	25 (25)	ELISA
m4–m8	[100]	2016	2, Canada, Milk before spray drying	Mean ± SD	21.95 ± 5.15	ELISA
m4–m8	[100]	2016	2, Canada, Milk before freeze drying	Mean ± SD	22.48 ± 5.84	ELISA
m1–m12	[93]	2020	26, Poland, Healthy	Mean ± SD	3.0 ± 2.89	ELISA
m13–m18	[93]	2020	35, Poland, Healthy	Mean ± SD	2.81 ± 2.74	ELISA
m19–m24	[93]	2020	32, Poland, Healthy	Mean ± SD	2.79 ± 2.41	ELISA
>m24	[93]	2020	23, Poland, Healthy	Mean ± SD	3.82 ± 3.05	ELISA
m3–m26	[94]	2013	5, Spain, Healthy, Unprocessed milk	Mean ± SD	22.9 ± 0.2	ELISA
m3–m26	[94]	2013	6, Spain, Healthy, Unprocessed milk	Mean ± SD	9.3 ± 0.5	ELISA
*	[52]	2013	8, Spain, Healthy, Untreated milk	Median (IQR)	10.67 (5.91–12.74)	Luminex
*	[52]	2013	8, Spain, Healthy, Pasteurized milk	Median (IQR)	6.37 (4.43–7.91)	Luminex
*	[49]	1977	>25, Cardiff, Untreated milk	Mean	100	RI
*	[49]	1977	>25, Cardiff, Heat-treated milk (56° 30 min)	Mean	100	RI
*	[50]	1978	19, Caucasian and Turkish women, Healthy (16–40)	Mean ± SD	4047 ± 1170	RI

SD, standard deviation of the mean; SEM, standard error mean; IQR, interquartile range; ELISA, enzyme-linked immunosorbent assay; N, nephelometry; RI, radial immunodiffusion; SRI, single radial immunodiffusion; IDQR, quantitative radial immunoassay; CVID, common variable immunodeficiency; g.a., gestational age. * Data are not specified.

**Table 5:** IgG in milk.

Breastfeeding Phase and Time	Study	Year	Population Characteristics N, Location, Particular Characteristics (Age)	Measure of Centrality and Spread	Concentration and Distribution (mg/L)	Analysis Method
**Colostrum**						
d0	[45]	1982	11, New Zealand, Healthy	Mean (range)	530 (150–1910)	RI
h24	[70]	2015	77, Brazil, Healthy	Mean (SD)	883 (515)	ELISA
d1	[75]	2011	60, Gabon, Healthy	Mean ± SEM	2000 ± 1000	N
d1–d2	[62]	1985	20, Moscow, Healthy (25.5)	Mean (SD)	314 (123)	SRI
d2	[78]	2006	31, Bangladesh, Healthy (18–35)	Mean ± SD	95 ± 24	ELISA
d3	[65]	2009	1, Brazil, mother with CVID	Value	19.5	ELISA
d3	[65]	2009	1, Brazil, mother with CVID	Value	1121	ELISA
d3	[45]	1982	11, New Zealand, Apparently healthy	Mean (range)	190 (80–460)	RI
d2–d3	[42]	2004	82, Brazil, Healthy (21–41)	Median (range)	28.0 (9–530.0)	ELISA
d1–d4	[82]	2013	11, Portugal, Healthy, Unprocessed milk	Mean ± SD	199 ± 10	ELISA
d2–d4	[66]	2005	36, Brazil, <32 w of g.a., Non-pasteurized milk	Mean ± SD	76 ± 38	RI
d2–d4	[66]	2005	36, Brazil, <32 w of g.a., Pasteurized milk	Mean ± SD	18 ± 26	RI
d2–d4	[66]	2005	32, Brazil, 32–36 w of g.a., Non-pasteurized milk	Mean ± SD	47 ± 42	RI
d2–d4	[66]	2005	32, Brazil, 32–36 w of g.a., Pasteurized milk	Mean ± SD	10 ± 20	RI
d2–d4	[66]	2005	33, Brazil, >37 w of g.a., Non-pasteurized milk	Mean ± SD	54 ± 37	RI
d2–d4	[66]	2005	33, Brazil, >37 w of g.a., Pasteurized milk	Mean ± SD	15 ± 23	RI
d3–d4	[62]	1985	20, Moscow, Healthy (25.5)	Mean (SD)	141 (50)	SRI
d1–d5	[109]	1992	14	Mean	80.4	RI
*	[50]	1978	24, Caucasian and Turkish women (16–40)	Mean ± SD	473 ± 238	RI
**Transition milk**						
d5–d6	[62]	1985	20, Moscow, Healthy (25.5)	Mean (SD)	56 (18)	SRI
d6	[45]	1982	11, New Zealand, Healthy	Mean (range)	30 (20–40)	RI
d7	[75]	2011	60, Gabon, Health, Term delivery	Mean ± SEM	1400 ± 600	N
d7	[65]	2009	1, Brazil, mother with CVID	Value	13.7	ELISA
d7	[65]	2009	1, Brazil, mother with CVID	Value	91	ELISA
d5–d10	[87]	2008	30, India, Term delivery, Pre-pasteurization milk	Mean (SD)	0.0055 (0.0013)	ELISA
d5–d10	[87]	2008	30, India, Term delivery, Post-pasteurization milk	Mean (SD)	0.0022 (0.0006)	ELISA
d7–d8	[62]	1985	20, Moscow, Healthy (25.5)	Mean (SD)	141 (50)	SRI
d5–d11	[88]	2016	90, China, Healthy urban mothers	Median (IQR)	22 (13)	ELISA
d14	[45]	1982	11, New Zealand, Healthy	Mean (range)	40 (20–200)	RI
w2	[91]	2020	51, Netherlands, Healthy, Term delivery	Median (IQR)	52.10 (39.10–76.42)	Luminex
Less w2	[89]	1992	16, Canada, Non-ultrasonic homogenized milk	Mean ± SD	6.6 ± 4.7	I
Less w2	[89]	1992	16, Canada, Ultrasonic homogenized milk (<45°)	Mean ± SD	5.7 ± 4.8	I
Less w2	[89]	1992	16, Canada, Ultrasonic homogenized milk (>55°)	Mean ± SD	2.8 ± 4.5	I
d8–d22	[95]	2019	36, Oregon, Preterm delivery	~Mean	22	ELISA
**Mature milk**						
d12–d30	[88]	2016	90, China, Healthy urban mothers	Median (IQR)	23 (12)	ELISA
d21	[75]	2011	60, Gabon, Health, Term delivery	Mean ± SEM	700 ± 300	N
d15–d30	[96]	2017	4, Mexico, Healthy (30), Unprocessed milk	~Mean	500	N
d15–d30	[96]	2017	4, Mexico, Healthy (30), Pasteurized milk (85°)	~Mean	400	N
m1	[105]	2018	36, Jordan, Healthy, Term delivery	Mean (SD)	103 (41.0)	ELISA
d22–d36	[109]	1992	14	Mean	46.9	RI
d42	[45]	1982	11, New Zealand, Healthy	Mean (range)	30 (20–50)	RI
m1–m2	[88]	2016	90, China, Healthy urban mothers	Median (IQR)	20 (14)	ELISA
w6	[91]	2020	51, Netherlands, Healthy, Term delivery	Median (IQR)	43.60 (32.64–57.71)	Luminex
d70	[45]	1982	11, New Zealand, Healthy	Mean (range)	20 (10–40)	RI
m3	[53]	2018	7, Alberta, Healthy	~Mean	12	ELISA
m3	[53]	2018	5, Alberta, Mothers with CD	~Mean	30	ELISA
m3	[53]	2018	11, Alberta, Mothers with UC	~Mean	20	ELISA
w12	[91]	2020	51, Netherlands, Healthy, Term delivery	Median (IQR)	43.60 (32.64–57.71)	Luminex
m4	[105]	2018	36, Jordan, Healthy, Term delivery	Mean (SD)	133 (48.9)	ELISA
m2–m4	[88]	2016	90, China, Healthy urban mothers	Median (IQR)	24 (15)	ELISA
m1–m6	[48]	1977	12, India, Well-nourished women	Mean ± SEM	29 ± 9.2	RI
m1–m6	[48]	1977	10, India, Under-nourished women	Mean ± SEM	58 ± 34.1	RI
d14–d150	[67]	2017	41, Spain, Healthy (>18)	Mean (IQR)	59.95 (48.73–90.51)	Luminex
d14–d150	[67]	2017	40, Ethiopia, Healthy (>18)	Mean (IQR)	96.09 (72.22–127.69)	Luminex
d14–d150	[67]	2017	41, USA/Washington, Healthy (>18)	Mean (IQR)	32.67 (19.35–44.60)	Luminex
m6	[105]	2018	36, Jordan, Healthy, Term delivery	Mean (SD)	145 (54.0)	ELISA
m6	[53]	2018	7, Alberta, Healthy	~Mean	350	ELISA
m6	[53]	2018	6, Alberta, Mothers with CD	~Mean	50	ELISA
m6	[53]	2018	7, Alberta, Mothers with UC	~Mean	50	ELISA
m4–m8	[100]	2016	2, Canada, Milk before spray drying	Mean ± SD	13.92 ± 0.80	ELISA
m4–m8	[100]	2016	2, Canada, Milk before freeze drying	Mean ± SD	19.59 ± 0.17	ELISA
m4–m8	[88]	2016	90, China, Healthy urban mothers	Median (IQR)	23 (14)	ELISA
m1–m12	[93]	2020	26, Poland, Healthy	Mean ± SD	14.71 ± 6.18	ELISA
m13–m18	[93]	2020	35, Poland, Healthy	Mean ± SD	14.82 ± 9.11	ELISA
m19–m24	[93]	2020	32, Poland, Healthy	Mean ± SD	15.60 ± 4.33	ELISA
>m24	[93]	2020	23, Poland, Healthy	Mean ± SD	18.95 ± 6.76	ELISA
m3–m26	[94]	2013	5, Spain, Healthy, Unprocessed milk	Mean ± SD	54.4 ± 2.2	ELISA
m3–m26	[94]	2013	6, Spain, Healthy, Unprocessed milk	Mean ± SD	13.5 ± 1.6	ELISA
*	[50]	1978	24, Caucasian and Turkish women (16–40)	Mean ± SD	234 ± 129	RI

SD, standard deviation of the mean; SEM, standard error mean; IQR, interquartile range; ELISA, enzyme-linked immunosorbent assay; N, nephelometry; RI, radial immunodiffusion; I, Immunodiffusion; SRI, single radial immunodiffusion; CVID, common variable immunodeficiency; CD, Crohn disease; UC, ulcerative colitis; g.a., gestational age. * Data are not specified.

**Table 6:** IgG subtypes in milk.

Breastfeeding Phase and Time	Study	Year	Population Characteristics N, Location, Particular Characteristics	Measure of Centrality and Spread	Concentration and Distribution (mg/L)	Analysis Method
***IgG1***						
**Colostrum**						
d2	[56]	1989	7, Colorado and Reykhavik	Mean ± SEM	2248.4 ± 531.8	ELISA
d3	[56]	1989	7, Colorado and Reykhavik	Mean ± SEM	539.8 ± 123.6	ELISA
d4	[56]	1989	7, Colorado and Reykhavik	Mean ± SEM	195.0 ± 83.2	ELISA
h96	[41]	2015	15, Spain, Preterm delivery	Mean (IQR)	87.80 (11.63–163.97)	Luminex
d1–d5	[109]	1992	14, California	Mean	37.2	RI
*	[52]	2013	6, Spain, Healthy, Untreated milk	Median (IQR)	102.61 (45.28–242.07)	Luminex
*	[52]	2013	3, Spain, Healthy, Pasteurized milk	Median (IQR)	157.85 (25.63–270.73)	Luminex
**Mature milk**						
d22–d36	[109]	1992	14, California	Mean	25.1	RI
d14–d56	[41]	2015	11, Spain, Preterm delivery	Mean (IQR)	10.36 (5.05–15.65)	Luminex
d49–d266	[56]	1989	11, Colorado and Reykhavik	Mean ± SEM	35.72 ± 4.40	ELISA
*	[52]	2013	3, Spain, Healthy, Untreated milk	Median (IQR)	36.70 (1.25–70.65)	Luminex
*	[52]	2013	2, Spain, Healthy, Pasteurized milk	Median (IQR)	16.20 (15.84–16.56)	Luminex
***IgG2***						
**Colostrum**						
d2	[56]	1989	7, Colorado and Reykhavik	Mean ± SEM	162.2 ± 59.6	ELISA
d3	[56]	1989	7, Colorado and Reykhavik	Mean ± SEM	38.0 ± 11.2	ELISA
d4	[56]	1989	7, Colorado and Reykhavik	Mean ± SEM	12.3 ± 0.4	ELISA
h96	[41]	2015	15, Spain, Preterm delivery	Mean (IQR)	68.04 (−2.92 to 139.00)	Luminex
d1–d5	[109]	1992	14, California	Mean	34.9	RI
**Mature milk**						
d22–d36	[109]	1992	14, California	Mean	19.6	RI
d14–d56	[41]	2015	11, Spain, Preterm delivery	Mean (IQR)	-	Luminex
d49–d266	[56]	1989	11, Colorado and Reykhavik	Mean ± SEM	4.18 ± 0.69	ELISA
***IgG3***						
**Colostrum**						
d2	[56]	1989	7, Colorado and Reykhavik	Mean ± SEM	113.9 ± 47.0	ELISA
d3	[56]	1989	7, Colorado and Reykhavik	Mean ± SEM	36.5 ± 10.1	ELISA
d4	[56]	1989	7, Colorado and Reykhavik	Mean ± SEM	14.7 ± 2.5	ELISA
h96	[41]	2015	15, Spain, Preterm delivery	Mean (IQR)	2.82 (0.98–4.65)	Luminex
d1–d5	[109]	1992	14, California	Mean	<3.4	RI
*	[52]	2013	4, Spain, Healthy, Untreated milk	Median (IQR)	16.45 (15.30–38.90)	Luminex
**Mature milk**						
d22–d36	[109]	1992	14, California	Mean	<1.6	RI
d14–d56	[41]	2015	11, Spain, Preterm delivery	Mean (IQR)	0.24 (0.11–0.37)	Luminex
d49–d266	[56]	1989	11, Colorado and Reykhavik	Mean ± SEM	1.31 ± 0.15	ELISA
***IgG4***						
**Colostrum**						
d2	[56]	1989	7, Colorado and Reykhavik	Mean ± SEM	14.7 ± 5.7	ELISA
d3	[56]	1989	7, Colorado and Reykhavik	Mean ± SEM	4.7 ± 1.0	ELISA
d2–d4	[68]	1983	27, Torrance	Mean (range)	4.6 (0.6–19)	RIA
d4	[56]	1989	7, Colorado and Reykhavik	Mean ± SEM	2.4 ± 0.4	ELISA
h96	[41]	2015	15, Spain, Preterm delivery	Mean (IQR)	0.98 (0.45–1.52)	Luminex
d1–d5	[109]	1992	14, California	Mean	4.9	RI
*	[52]	2013	10, Spain, Healthy, Untreated milk	Median (IQR)	649.80 (474.63–984.41)	Luminex
*	[52]	2013	9, Spain, Healthy, Pasteurized milk	Median (IQR)	530.67 (410.95–902.78)	Luminex
**Mature milk**						
d22–d36	[109]	1992	14, California	Mean	4.2	RI
d14–d56	[41]	2015	11, Spain, Preterm delivery	Mean (IQR)	0.29 (0.12–0.46)	Luminex
d49–d266	[56]	1989	11, Colorado and Reykhavik	Mean ± SEM	0.516 ± 0.109	ELISA
*	[52]	2013	8, Spain, Healthy, Untreated milk	Median (IQR)	517.23 (236.18–701.76)	Luminex
*	[52]	2013	8, Spain, Healthy, Pasteurized milk	Median (IQR)	365.50 (324.55–410.95)	Luminex

SD, standard deviation of the mean; SEM, standard error mean; IQR, interquartile range; ELISA, enzyme-linked immunosorbent assay; RI, Radial immunodiffusion; RIA, radioimmunoassay; SRI, Single radial immunodiffusion; * Data are not specified.

**Table 7:** IgE and IgD in milk.

Breastfeeding Phase and Time	Study	Year	Population Characteristics N, Location, Particular Characteristics (Age)	Measure of Centrality and Spread	Concentration and Distribution (mg/L)	Analysis Method
***IgE***						
**Colostrum**						
d0–d4	[54]	1982	15, California (16–41)	Range	0.0012–0.014	RIA
d2–d4	[51]	1996	39, Sweden, Atopic and non-atopic mothers	Mean	0.0003	PRIST
*	[52]	2013	10, Spain, Healthy, Untreated milk	Median (IQR)	0.67 (0.44–1.12)	Luminex
*	[52]	2013	7, Spain, Healthy, Pasteurized milk	Median (IQR)	1.17 (0.87–1.48)	Luminex
**Mature milk**						
m3	[53]	2018	7, Alberta, Healthy	~Mean	0.0011	ELISA
m3	[53]	2018	5, Alberta, Mothers with CD	~Mean	0.0011	ELISA
m3	[53]	2018	11, Alberta, Mothers with UC	~Mean	0.0018	ELISA
m6	[53]	2018	7, Alberta, Healthy	~Mean	0.001	ELISA
m6	[53]	2018	6, Alberta, Mothers with CD	~Mean	0.0011	ELISA
m6	[53]	2018	7, Alberta, Mothers with UC	~Mean	0.0012	ELISA
*	[52]	2013	3, Spain, Healthy, Untreated milk	Median (IQR)	0.43 (0.21–0.85)	Luminex
*	[52]	2013	7, Spain, Healthy, Pasteurized milk	Median (IQR)	0.43 (0.20–0.81)	Luminex
***IgD***						
**Colostrum**						
d1–d5	[55]	1985	31, California	Mean	0.358	PDSP
d0–d4	[54]	1982	39, California (16–41)	Mean (range)	0.413 (0.02–20)	RIA

SD, standard deviation of the mean; SEM, standard error mean; IQR, interquartile range; ELISA, enzyme-linked immunosorbent assay; N, Nephelometry; T, Turbidimetry; RI, Radial immunodiffusion; SRI, Single radial immunodiffusion; PRIST, paper disc radioimmunosorbent test; RIA, radioimmunoassay; PDSP, paper disc solid phase; CD, Crohn disease; UC, ulcerative colitis; g.a., gestational age. * Data are not specified.

## Data Availability

Not applicable.
